# Comparison of Flexural Strength and Wear of Injectable, Flowable and Paste Composite Resins

**DOI:** 10.3390/ma17194749

**Published:** 2024-09-27

**Authors:** Hadi Rajabi, Michael Denny, Kostas Karagiannopoulos, Haralampos Petridis

**Affiliations:** Department of Prosthodontics, University College London Eastman Dental Institute, Rockefeller Building, 21 University Street, London WC1E 6DE, UK; haddent@hotmail.co.uk (H.R.); michaeldenny@outlook.com (M.D.); kostas.karagiannopoulos@gmail.com (K.K.)

**Keywords:** dental composite resin, wear, flexural strength, injectable resin, flowable resin, composite resin paste, highly filled flowable resin, dental materials, polymers, mechanical properties

## Abstract

(1) Objectives: This study investigated and compared the wear and flexural strength of two highly filled (injectable), one flowable and one paste composite. (2) Methods: Two highly filled flowable composites (G-aenial Universal Injectable and Beautifil Plus F00), a paste composite (Empress Direct) and a conventional flowable (Tetric EvoFlow) were tested. A two-body wear test was carried out using 10 disc-shaped samples from each group, which were subjected to 200,000 wear machine cycles to simulate wear, followed by Scanning Electron Microscope analysis. Flexural strength was tested using a three-point bend test using 15 beam samples for each of the four groups. Values were statistically compared using one-way analysis of variance (ANOVA) for flexural strength and a Kruskal–Wallis test for wear. (3) Results: The median volume loss for G-aenial Universal Injectable and Beautifil Plus F00 was statistically lower than that of both Empress Direct and Tetric EvoFlow. For flexural strength the two highly filled flowable composites both exhibited statistically higher mean flexural strength values compared to Empress Direct (*p* < 0.004) and Tetric Evoflow (*p* < 0.001). There were no statistically significant differences in the values of wear and flexural strength between the two highly filled flowable composites. (4) Conclusions/significance: Highly filled flowable composite resins with nano filler particles outperformed a conventional flowable and a paste composite resin in terms of wear resistance and flexural strength, and may be suitable to use in occlusal, load-bearing areas.

## 1. Introduction

Composite resins have an integral place in the hands of the restorative dentist and have become the material of choice for direct restorations owing to their improved aesthetics, advances in wear and mechanical properties, as well as repairability [1,2]. Despite their success they still experience failures mainly because of secondary caries and fractures and are still regarded as being technique sensitive [2]. The main two families of composite resins in terms of application are the packable pastes and flowable composites. Flowable composite resins were first introduced in 1996, defined as the less viscous resin composite [3]. Historically, flowable composite resins were not recommended for high-stress-bearing areas, larger cavities, or occlusal cavities; however, recently flowable composite resins with a higher filler content and modified filler size have been developed [4]. These materials have a lower modulus of elasticity and improved mechanical properties and wear resistance, allowing for a far larger range of applications including restorations of posterior teeth [5]. The newly designed highly filled flowable composite resins may exhibit more resistance to crack propagation, resulting in better wear characteristics than the widely used traditional nanohybrid resin composites [6]. This is due to some novel changes in the filler size and composition, filler silanisation, and production procedures [7,8]. Such highly filled flowable composite resin materials have also been introduced to the market as “injectable composites”, examples of which being the G-aenial universal injectable composite (GC Corporation, Tokyo, Japan) and Beautifil Flow Plus (Shofu, Kyoto, Japan) (also marketed as Shofu Beautifil injectable outside of the European Union). Injectable composites are highly filled, low-viscosity nano-hybrid restorative materials and the advantage of the injectable technique is the ability to replicate the diagnostic tooth morphology without relying on free-hand application, as with traditional composite resin materials [1,6,9].

In a clinical study, an injectable composite showed comparable clinical effectiveness to the conventional paste composite in posterior restorations over 36 months [5]. The study findings highlighted several benefits associated with using injectable composites, including easier handling, improved cavity wall adaptation, and reduced time required for placing the restoration [5]. A group of six highly filled flowable composite resins exhibited higher flexural properties and wear resistance compared to two conventional composite resin pastes [6]. Similarly, in a study of handling and mechanical properties of low viscosity composite resins, it was evident that some exhibited excellent flexural properties and wear resistance [10]. When the effects of acidic beverages on the surface roughness, microhardness, flexural strength and elastic modules were evaluated for a micro-hybrid, bulk-fill and injectable composites, it was evident that the highly filled “injectable” exhibited the highest mean flexural strength values and, interestingly, when exposed to short- and long-term immersion cycles it exhibited a flexural strength value above ISO 4049/2019 standards [11], which is promising for clinical use [12]. In a comparison of the wear and flexural characteristics of highly filled flowable composites with their respective paste composites produced by the same manufacturer, it was concluded that the former exhibited improved performance [13].

A difficulty when interpreting the literature is that some studies have used predecessor materials to the current materials in the market and that different terminologies have been used such as “flowable”, “high filled flowable”, “bulk fill” and “injectable” composites. Therefore, the highly filled flowable composites branded as injectable composites have not been extensively researched, leading into a gap in knowledge about their physical properties and potential suitability for restorative procedures in areas subjected to occlusal loading. The injectable technique is mainly used to restore anterior fractured or worn teeth but, many times, these restorations will still be subject to increased occlusal loading. Conventional paste composite resins cannot be used with the injectable technique and traditional flowable composite resins are inherently weaker materials [1,4,5,6,9,14]. Some highly filled flowable composites have been shown to have inferior mechanical properties to paste composites [14], whereas others have been shown to perform as well as, if not better than, paste composites [6]. Therefore, there is a need to investigate some novel highly filled “injectable” composites currently on the market, and to clearly define their clinical indications.

The aim of this study was to compare the flexural strength and wear resistance characteristics of two highly filled injectable composite resin materials (G-aenial Universal Injectable, GC Corporation, Tokyo, Japan, and Beautifil Flow Plus F00, Shofu, Kyoto, Japan) with those of two conventional composite resin materials: a nano-hybrid flowable material (Tetric EvoFlow, Ivoclar Vivadent, Schaan, Liechtenstein) and a nano-hybrid composite resin paste (IPS Empress Direct Enamel, Ivoclar Vivadent, Schaan, Liechtenstein). 

The null hypothesis was that there would be no statistically significant difference in the flexural strength and wear resistance values between these forms of dental composite resin materials (paste, flowable and injectable).

## 2. Materials and Methods

Four composite resin materials formed the four groups of the study. Two injectable/highly filled flowable composite resins: Beautifil Flow Plus F00 (Shofu, Kyoto, Japan), G-aenial Universal Injectable (GC corporation, Tokyo, Japan), and a flowable composite resin, Tetric EvoFlow (Ivoclar Vivadent, Schaan, Liechtenstein) were used in this study. As a control group, one resin composite paste—IPS Empress Direct Enamel (Ivoclar Vivadent, Schaan, Liechtenstein)—was used (Table 1).

### 2.1. Testing of Wear Resistance

In this study, a two-body wear test was used to assess wear resistance. Four groups of specimens were used, representing the four resin composites tested (N = 10). The sample size was determined based on a pilot study and subsequent sample size estimation using G8Power software (v3.1.9.7, Dusseldorf, Germany) [15]. Disk-shaped specimens, each measuring 10 mm in diameter and 2 mm in depth, were created from each composite resin material using milled moulds. These moulds were initially designed with the aid of Autodesk MeshMixer software (v3.5.474; Autodesk, Inc., San Rafael, CA, USA), before undergoing a milling process. The moulds were designed with a view to be fitted in the chewing simulator. The selected materials were then polymerised in accordance with both the ISO 4049:2019 standards [11] and the specific instructions provided by the respective manufacturers. The composite resin was placed into the mould and packed using an acetate sheet and a glass slab. It was pressed to achieve uniform thickness, eliminate any extra material, avoid the formation of an oxygen inhibition layer and creating a flat surface. Each sample was polymerised for 30 s using a10 mm tip diameter LED curing unit (Bluephase G4; Ivoclar Vivadent AG, Schaan, Liechtenstein) with 385–515 nm wavelength and 1200 mW/cm^2^ light intensity, which was controlled periodically using the radiometer on the light cure machine. Lastly, any extraneous material, or ‘flash’, present around the edges was delicately removed using a number 12 surgical scalpel blade. After the preparation, the specimens were placed in distilled water and kept in an incubator for 24 h at a temperature mirroring the average human body temperature of 37 °C. The specimens were labelled and subsequently positioned in chewing simulator holders to facilitate the wear test.

A chewing simulator, Mechatronik CS-4.4 (SD Mechatronik, Germany), was used to carry out a two-body wear test, utilising chewing cycles by means of linear two-axis motion. Steatite balls with a diameter of 6 mm (Mechatronik, Feldkirchen-Westerham, Germany) were used as antagonists and inserted into milled moulds, which were subsequently positioned in aluminium sample holders. New steatite balls were assigned to each group. The specimen distribution within the chewing simulator chambers was executed randomly. Four samples underwent wear testing at a time. The loading parameters incorporated a vertical stroke: ↑ 2 mm ↓ 2.5 mm; vertical speed: ↑ 60 mm/s ↓ 20 mm/s; horizontal stroke: 2 mm; horizontal speed: 20 mm/s; a frequency of 1.4 Hz; and a load of 49 N, facilitated by the chewing simulator (Mechatronik CS-4.4, SD Mechatronik, Germany). The testing was carried out for 200,000 cycles under dry conditions, emulating approximately one year of clinical performance [16]. This was repeated until all 40 samples were tested. 

**Figure 1:**
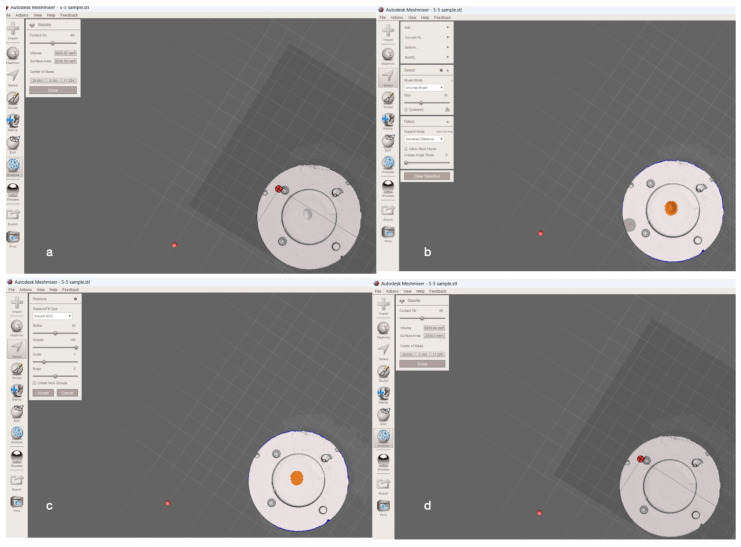
The total volumetric loss measurement using the Autodesk MeshMixer software. (**a**) Initial view; (**b**) stability function to determine the initial volume; (**c**) erase and fill function; (**d**) stability function to measure the wear volume loss.

The specimens were digitally scanned using a digital scanner (Zirkonzahn S900 ARTI Scanner, Tyrol, Italy). The scans (.stl files) were then imported into Autodesk MeshMixer software (v3.5.474; Autodesk, Inc., San Rafael, CA, USA) to measure the total volumetric wear loss. As completely flat-surfaced composite discs were used, a single scan method was utilized, which eliminates the need for a separate baseline scan by measuring wear merely using the three-dimensional scan data of the worn sample [17]. Contrary to the conventional techniques that require the merging of datasets of the scanned pre-and post-wear tests, this approach saves time and reduces potential errors associated with repeated scans and overlaying procedures [17]. The “Stability” function was accessed to record the 3D model’s initial volume. All portions of the worn area, including adjacent unworn sections, were selected. The “Erase & Fill” function was employed to revert the worn region to its original state. The volume was then reassessed using the “Stability” function, and the wear volume loss was calculated by comparing the initial and restored model volumes (Figure 1).

Scanning Electron Microscope (SEM) analysis was also carried out for one specimen/group. The specimens were coated with Gold-Palladium (Au-Pd) alloy and transferred to the SEM (Zeiss Sigma 300 VP Microscope, Carl Zeiss AG, Oberkochen, Germany) for the purpose of image detection and analysis at various magnification levels. 

For statistical analysis, IBM SPSS software version 28.0.1 (SPSS/IBM, Armonk, NY, USA) was employed. Data from Excel worksheets were imported for analysis. The performed statistical analysis included both the Kruskal–Wallis test and the Bonferroni test. 

### 2.2. Testing of Flexural Strength

A three-point bend test was used to measure bi-axial flexural strength for the four types of composite resins tested. Fifteen samples of each resin composite were manufactured using a stainless-steel split mould, which has an internal rectangular recess to produce samples of 25 mm length/2 mm width/2 mm depth. The sample size was determined based on a pilot study and subsequent sample size estimation with G8Power software (v3.1.9.7, Dusseldorf, Germany) [15]. The composite resin was inserted in one single increment in the mould. A clear acetate strip was placed over the composite-filled mould, followed by a glass slide and finally a clamp to apply pressure to the mould to squeeze out excess material. The selected materials were then processed and polymerised in accordance with both the ISO 4049:2019 standards [11] and the specific instructions provided by the respective manufacturers. Curing was achieved using a BluePhase LED light curing unit (Bluephase G4; Ivoclar Vivadent AG, Schaan, Liechtenstein) with 385–515 nm wavelength and 1200 mW/cm^2^ light intensity, with 20 s curing applied to five overlapping sites to avoid the risk of differential curing within the sample, which was repeated for both sides of the sample. Five sites were used for each side due to the tip diameter of the light curing unit being 10 mm. The output of the curing light was tested after curing 5 samples using the built-in radiometer. 

The polymerised samples were removed and placed into a water bath for 15 min as per the ISO 4049 guidance [11]. After 15 min the samples were removed and, using 8× magnification, the excess was removed using a scalpel blade. The samples were inspected for surface defects and voids again under 8× magnification, and measured using digital calipers to ensure they met the required dimensions of 25 × 2 × 2 mm. The samples were placed into a water bath containing distilled water at 37 °C, for a period of 24 h from initial light curing. After a period of 24 h the samples were removed from the water bath and tested using the Shimadzu Universal testing machine (Shimadzu Corporation, Kyoto, Japan) at a crosshead speed of 1 mm per minute. The Shimadzu Universal testing machine was programmed to load the beam at a cross-head speed of 1 mm per min, as per ISO 4049:2019 guidelines (0.75 +/− 0.25 mm/min) [11]. The samples were loaded and the load at failure was recorded for each sample in Newtons, using the Trapezium software system X (10) (Trapezium X, Shimadzu, Kyoto, Japan). The flexural strength values in MPa were calculated using the equation obtained from ISO 4049:2019 [11].

$$\sigma =\frac{3Fl}{2bh^{2}}$$

*σ*—Flexural strength in megapascals (MPa)

*F*—Maximum load, in Newtons, exerted on the specimen

*l*—Distance, in millimeters, between the supports, accurate to 0.01 mm

*b*—Width, in millimeters, at the centre of the specimen measured prior to testing

*h*—Height, in millimeters, at the centre of the specimen measured prior to testing

The *F* value in Newtons was obtained from the Trapezium X software linked to the Shimadzu Universal testing machine; *l* was 20 mm, *b* and *h* were each 2 mm.

For statistical analysis, IBM SPSS software version 28.0.1 (SPSS/IBM, Armonk, NY, USA) was employed. The flexural strength test mean values were normally distributed and therefore, a one-way ANOVA was used to statistically compare the groups. To assess the difference between the mean values, post hoc tests were completed, and Bonferroni adjusted *p*-values were produced to compare the difference between the mean scores of groups.

## 3. Results

### 3.1. Wear Resistance

**Figure 2:**
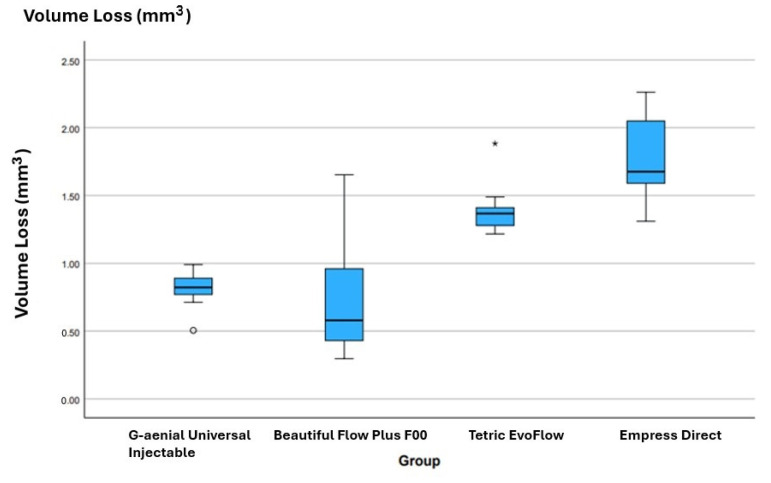
Box and whisker plot showing the total volumetric wear loss per group after 200 K load cycle. Line indicates median and whisker ends indicate 25th and 75th quartile values. Asterisk indicates outlier value.

The median values and the 25th quartile range of total volumetric loss are shown in Figure 2. Due to the non-normal distribution of the data, a non-parametric Kruskal–Wallis test was conducted, and showed a statistically significant difference between the groups (*p* < 0.001). Therefore, the first null hypothesis regarding wear resistance was rejected. 

In order to determine the groups with differences, non-parametric pairwise tests were carried out, that compared each pair of groups (Table 2). The *p*-value (indicated in the final column) was adjusted for multiple testing to reduce the probability of encountering spuriously significant results. Overall, the median volume loss was not statistically different between Beautifil Flow Plus F00 and G-aenial Universal Injectable. Both showed statistically reduced volume loss compared to the paste Empress Direct Enamel and lower values compared to Tetric EvoFlow.

**Figure 3:**
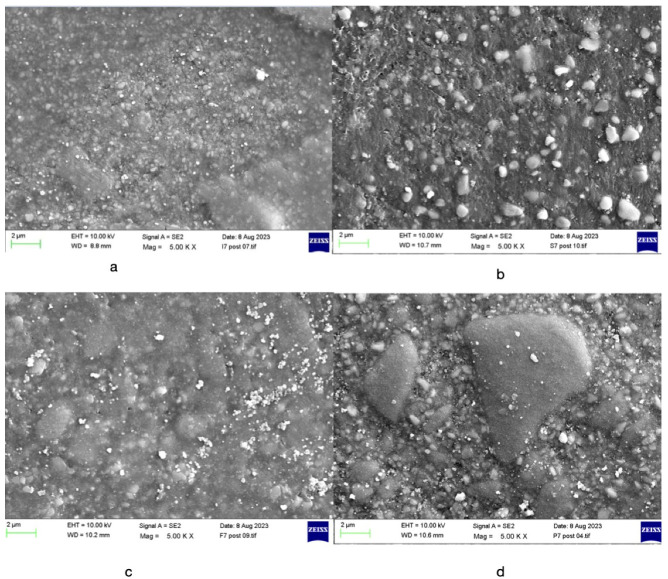
SEM analysis for (**a**) G-aenial Universal Injectable, (**b**) Beautifil Flow Plus F00, (**c**) Tetric EvoFlow, (**d**) IPS Empress Direct. All views are at 5K magnification.

The SEM analysis of the four composite resin materials, after 200,000 cycles of the localised wear test, can be viewed in Figure 3. The SEM analysis showed that the surfaces of G-aenial Universal Injectable appeared smoother, with more regular-shaped filler particles. Tetric EvoFlow revealed instances where fillers had become dislodged, leading to dark voids in the SEM images. Beautifil Flow Plus F00 displayed signs of fractures between the irregularly shaped polymerised filler and the matrix resin. IPS Empress Direct appeared less smooth, with irregularly shaped filler particles of larger sizes.

### 3.2. Flexural Strength

**Figure 4:**
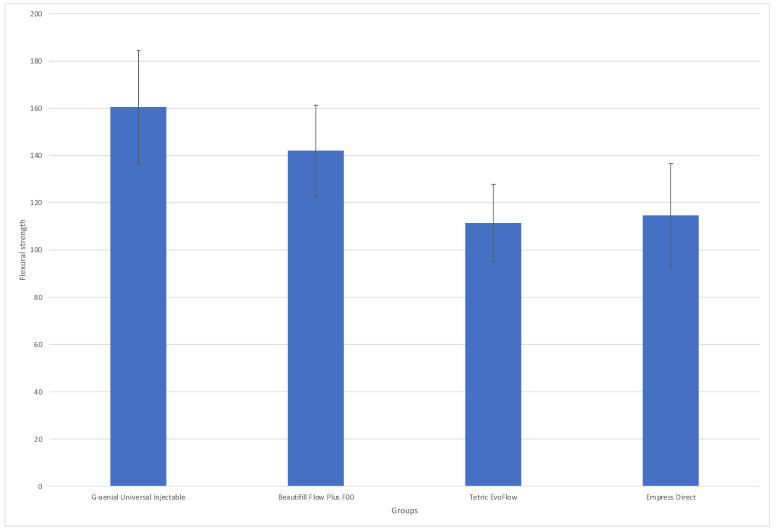
Box and whisker diagram showing flexural strength values (MPa) for each group. Line indicates mean and whisker ends indicate minimum and maximum values along with some outlier values as points.

The descriptive statistics of the flexural test are shown in Table 3 and Figure 4. One-way ANOVA showed that the mean values of the four groups were statistically different (*p* < 0.001), so the null hypothesis regarding flexural strength was rejected.

The highest mean flexural strength was observed for the G-aenial universal injectable group at 160.49 MPa and Beautiful Flow Plus at 141.97 MPa. The post hoc analysis showed that there was no statistically significant difference between the mean values of GC injectable and Beautiful Flow Plus (*p* ≥ 0.05) but they both exhibited statistically higher mean flexural strength values compared to Empress Direct (*p* < 0.004) and Tetric Evoflow (*p* < 0.001). The latter two groups did not exhibit a statistically significant difference in mean values (*p* ≥ 0.05).

## 4. Discussion

Highly filled, flowable composite resins possess a lower elastic modulus and a greater capacity to absorb stress, broadening their potential applications to encompass a range of restoration classifications and direct composite resin veneers [6,18].

Wear resistance of composite resins can be discerned by observing the impact of the filler content, the inherent characteristics of the matrix, and the efficacy of the bond mediated by the coupling agent between the filler and the matrix [19]. Over time, a diverse range of filler systems, monomer configurations, and coupling agents have been innovated to enhance both the wear resistance and mechanical properties of composite resins [20].

The wear resistance of composite resin materials is influenced by the cumulative damage caused by repeated stress (fatigue). Hence, applying in vitro fatigue to composite resins enhances the clinical applicability of the results [21]. This is achieved by employing simulators that apply cyclic loading on the specimens, to mimic the challenges present in the oral environment. The SD Mechatronic chewing simulator utilised in the current study is an example of such a device. The chewing forces employed during simulation are typically around 5 kg (49 N) as the mean chewing force during normal function [22]. In this study, steatite abraders were used, as they have demonstrated a wear behaviour akin to natural teeth [23]. 

The results of this study showed that the two highly filled flowable composite resins (Beautifil Flow Plus F00 and G-aenial Universal Injectable) displayed a statistically significant greater wear resistance compared to a conventional nanohybrid composite resin, IPS Empress Direct Enamel. The findings from this study align with the two-body wear testing carried out by Shinkai et al. [24], where they examined the impact of cyclic loading on the surface characteristics of four distinct flowable resin composites, using a universal paste as the control. The results are also in agreement with other studies [6,10] looking at the characteristics of these novel composite resins. Furthermore, the SEM images in this study exhibited a smoother surface for the resin with the higher filler content and smaller particle size. The average filler size and filler volume have historically been linked to the wear characteristics of resin composites [24,25]. Composite resins with a higher proportion of filler and smaller particle sizes tend to exhibit reduced wear [25]. This corresponds with the findings of this study, where the two injectable composites with a higher weight percentage of inorganic filler content exhibited superior performance compared to the Tetric EvoFlow, which had a lower filler content. However, they also outperformed the paste composite, which had a higher percentage of filler content; so it seems that there are now further aspects that affect performance which have to do with filler size, distribution and bonding to the resin matrix, which are yet to be evaluated. Enhanced wear resistance might be attributed to the reduced inter-particle spacing in small-particle fillers within contact-free regions or improved silane bonding between fillers and the organic matrix. This phenomenon could be due to the technology employed, ensuring that smaller filler particles are densely packed. As a result, the resin in between is protected from further wear caused by adjacent particles [13].

This disparity might also originate from the fact that each of the examined composite resins possesses a distinct resin matrix. Variations in resin matrices give rise to diverse viscosities, molecular weights, and structural scaffolds. For instance, the Bis-GMA monomer is known for its elevated viscosity and rigid structural backbone [26].

In this study, a three-point bend test was used to measure bi-axial flexural strength for the four groups tested. Criticisms of the three-point bend test often cited in the literature include the relatively large size of the specimens, which require multiple exposures to light curing and could result in sites of differentially cured material, and may not reflect the size of dental restorations. The beam shape means that specimens are more susceptible to edge defects, which can act as sites of stress concentration, leading to premature fracture [27]. In a study comparing a micro-hybrid and nano-fill composite resin by three-point and four-point bend tests, higher flexural strength scores were evident in the three-point bend test due to the smaller area of the beam being under load and therefore less likely to incorporate critical flaws within the sample, that would lead to failure compared to the four-point bend test, which loads a larger area of the sample [28]. Another limitation relates to the fact that mechanical failure of composite restorations occurs due to the propagation of sub-surface imperfections after repeated insults over a period of time; hence, a cyclic fatigue test may be more appropriate in determining mechanical strength [29]. Furthermore, a limitation of this study was that the two-body wear testing was done in dry conditions, which may not be as clinically relevant as testing in wet conditions. Water offers a lubricating function and can reduce wear by removing debris [30,31] but at the same time testing in wet conditions can lead to hygroscopic expansion and hydrolytic degradation due to water absorption, which may decrease the physical and mechanical properties of composites [30], leading to increased wear.

The results of this study showed a statistically significant difference in the mean flexural strength values between the two highly filled flowable composite resins, and the paste and conventional flowable resins. The lack of significant difference between the paste and the conventional flowable could be associated with the difficulty in fabricating samples with the paste composite and the potential for voids within the samples. However, the mean flexural strength value for Empress Direct paste was 114.69 MPa, which is not too dissimilar from the value reported by the manufacturer of 120 MPa [32]. The paste composite used in this study has relatively low flexural strength values compared to some other pastes on the market; however, it is a commonly used paste material and therefore was selected for comparison. Another important consideration is that the paste had an enamel formulation and the manufacturer’s data suggest that the flexural strength value of this is higher than that of the dentin shade [32]. However, the manufacturer states that the dentin shade contains prepolymer fillers and larger 0.7 µm barium fillers to ‘improve the strength’ of the dentin shade. This contrasts with the smaller 0.4 µm barium fillers and the lack of prepolymer fillers in the enamel shade. Therefore, the flexural strength values reported in this study are more specific to the shade and the enamel type chosen. The mean value for flexural strength for Tetric EvoFlow was 111.39 MPa and, according to the manufacturer’s data, the value is 114 MPa [33]. These values exceed the values stated in the ISO 4049:2019 [11] standards for minimum flexural strength values required for occlusal surfaces, which is interesting since these are not materials that would typically be advocated for sites of occlusal loading and perhaps demonstrates how flowable materials have undergone a period of evolution in properties. 

The mean value for flexural strength for G-aenial Universal Injectable was 160.49 MPa and according to the manufacturer’s data the value is 173 MPa [34].

A recent study [6] compared mechanical properties between two injectable composites G-aenial universal flo (GC Corporation, Tokyo, Japan) and Beautifil Flow Plus F00 (Shofu, Kyoto, Japan) and two paste composites: Clear-fill APX (Kuraray, Tokyo, Japan) and Filtek supreme ultra (3M, St. Paul, MN, USA) [6]. The authors reported that Clear-fill APX had the highest flexural strength value of 180.1 MPa, the G-aenial Universal Flo yielded 154.9 MPa and the Beautifil Flow Plus F00 was much lower at 116.2 MPa. G-aenial Universal Flo is the predecessor material to the G-aenial Universal Injectable and is a highly filled flowable material with an identical filler content per weight, which could be regarded as having some of the properties of G-aenial universal injectable.

With respect to the Beautifil Flow Plus F00 (Shofu, Kyoto, Japan), the mean flexural strength value in this study exceeds that reported by the manufacturer and in the paper by Imai et al. [6,35]. 

The findings of this study are in line with other studies comparing flexural strength between flowable and paste composites or bulk fill composites [6,10,13]. It was also suggested that injectable composites may be indicated for thin occlusal veneers as thermomechanical cyclic loading influenced an injectable composite less than a milled resin-based material [18]. In a failure mode study [34] of occlusal overlays with fatigue testing of 0.5 mm occlusal veneers, G-aenial Universal Injectable was compared to a hybrid paste composite and a lithium disilicate ceramic as controls. The study revealed that the injectable composite was as reliable as lithium disilicate up to a year (250,000 cycles) at 100 N load level [36]. At load levels higher than 100 N, lithium disilicate outperformed the injectable composite [36]. Fatigue testing may be a more clinically relevant laboratory test than wear and flexural strength testing, and further research is needed to investigate fatigue testing for different thickness of occlusal veneers with injectable composites.

A 36-month clinical study comparing an injectable/highly filled flow (G-aenial universal Flo, GC Corporation, Tokyo, Japan) to a conventional paste—Estelite sigma quick (Tokuyama, Tokyo, Japan)—for posterior restorations demonstrated comparable clinical effectiveness between the two materials [5]. These short-term clinical results support the apparent good mechanical properties of these novel composite resin materials demonstrated in this study. Therefore, these materials might be considered suitable for use in dental restorations within high load-bearing areas. However, these results must be interpreted cautiously, considering that in vivo wear and mechanical failures might differ due to various biological, chemical, and physical challenges in the oral environment. It would be valuable to correlate these findings with long-term clinical observations and further investigate the underlying failure modes.

## 5. Conclusions

Within the limitations of this comparative in vitro study, in which geometric samples were used instead of real restorations, it could be concluded that the two highly filled “injectable” composite resins demonstrated improved wear resistance and flexural strength compared to a conventional flowable and a paste composite resin. The null hypotheses that there would be no statistically significant difference in the flexural strength and wear resistance in these four groups’ dental composite resins were thus rejected.

The results of this study highlighted the possible suitability of these highly filled flowable composite resins to be used in occlusal load-bearing areas; however, further research is indicated to assess fatigue stress behavior, as well as clinical performance of these novel composite resin materials.

## Figures and Tables

**Table 1:** Composite resins used in this study.

Group	Material	Composition-Resin Matrix	Composition-Filler	Filler wt./vol	Type	Manufacturer	Shade	Batch/Expiry
1	Tetric Evoflow	Bis-GMA, Urethane dimethacrylate,	Decandioldimethacrylate Barium glass filler, Ytterbium trifluoride, Mixed oxide, Highly dispersed silica Prepolymers	57.5/30.7	Nanohybrid flowable	Ivoclar Vivadent, Liechtenstein	A2	Z052D7/ 19/01/2027
2	Empress Direct Enamel	Bis-GMA, UDMA, TCDDMA,	Barium glass filler, mixed oxide, Ba-Al-fluorosilicate glass Mean size 550 nm range (40 nm–3 μm)	78.1/(52–59)	Nanohybrid paste	Ivoclar Vivadent, Liechtenstein	A2E	Z037HP/ 24/08/2025
3	Shofu Beautifil flow plus F00	Bis-GMA TEGDMA resin	Multifunctional glass filler and S-PRG filler based on fluroboroaluminosilicate glass. Particle size range: 0.01–4.0 μm Mean particle size: 0.8 μm DL-Camphorquinone	67.3/47	Nanohybrid injectable	Shofu Inc., Kyoto, Japan	A2	052316/ 30/04/2026
4	G-aenial Universal injectable	Monomers: dimethacrylate rmonomers;	barium glass, silica; photoinitiator	69/50	Nanofilled injectable	GC Tokyo, Japan	A2	2212131 12/12/2025

Bis-GMA: bisphenol-A glycidyl dimethacrylate; TEGDMA, triethylene glycol dimethacrylate; UDMA, urethane dimethacrylate; TCDMA: tricyclodocane dimethanol dimethacrylate.

**Table 2:** Pairwise comparisons of the four groups.

Composite	Test Statistic	Standard Error	Standard Test Statistic	Significance	Adjusted Significance (a)
Beautifill-G-ænial	2.600	5.228	0.0497	0.0619	1.000
Beautifill-Tetric	−16.000	5.228	−3.060	0.002	0.013
Beautifill-Empress	−23.800	5.228	−4.552	<0.001	0.000
G-ænial-Tetric	−13.400	5.228	−2.563	0.010	0.062
G-ænial-Empress	−21.000	5.228	−4.055	<0.001	0.000
Tetric-Empress	−7.800	5.228	−1.492	0.136	0.814

**Table 3:** Mean flexural strength and standard deviations of the four tested groups.

Composite	Mean Flexural Strength (MPa)	Standard Deviation	95% Confidence Interval for Lower Bound	95% Confidence Interval for Upper Bound	Minimum	Maximum
Tetric Evoflow	111.38	16.43	102.28	120.48	81.30	148.95
Empress Direct	114.68	21.90	102.55	126.81	62.93	147.30
Beautifil Flow PlusF00	141.97	19.33	131.26	152.67	96.11	174.83
GC injectable	160.49	24.02	147.18	173.79	117.41	217.80

## Data Availability

The original contributions presented in the study are included in the article, further inquiries can be directed to the corresponding author.
